# Study on genotoxicity, oxidative stress biomarkers and clinical symptoms in workers of an asbestos-cement factory

**DOI:** 10.17179/excli2015-469

**Published:** 2015-09-28

**Authors:** Azam Afaghi, Shahrbanoo Oryan, Kobra Rahzani, Mohammad Abdollahi

**Affiliations:** 1 Department of Biology, Science and Research Branch, Islamic Azad University, Tehran, Iran; 2 Arak University of Medical Science, Arak, Iran; 3 Department of Toxicology and Pharmacology, Faculty of Pharmacy and Pharmaceutical Sciences Research Center, Tehran University of Medical Science, Tehran, Iran

**Keywords:** asbestos, plant, oxidative stress, DNA damage, malondialdehyde, total thiol molecule, total antioxidant capacity

## Abstract

The purpose of the present study was to investigate whether the markers of oxidative stress could be altered in workers exposed to asbestos. A comparative cross-sectional research was conducted in a group of 50 subjects exposed to asbestos and another group of 50 with the same age and sex unexposed to asbestos. Malondialdehyde (MDA), total thiol molecule (TTM), total antioxidant capacity (TAC), and DNA damage, were measured in the blood samples of workers and controls. Compared to the control group, the workers showed higher blood levels of DNA damage (P=0.0001) and MDA (P=0.0001). The workers showed lower TTM (P=0.02) as compared with the control group. There was no considerable difference on the level of TAC (P=0.1) between the groups. The workers indicated clinical symptoms such as breathlessness, phlegm, coughing and wheezing. There was a positive correlation between levels of 8-OHdG and MDA of asbestos workers and the smoking status suggesting the negative role of smoking.

## Introduction

Asbestos is a group of silicate minerals consisting of fibers, found naturally in the environment and also produced during the process of crushing and processing in certain industries (Kakooei and Kakooei, 2007[15]). Based on the structure, asbestos is divided into two categories of amphibole and serpentine. Amphibole category includes amosite, tremolite, anthophylite, actinolite and crocidolite. The serpentine family contains only chrysotile (Ryu et al., 2012[38]). Asbestos fiber is odorless, tasteless, insoluble in water, and also does not evaporate. It is resistant against heat, flame and chemical and doesn’t disappear by biological methods (Ryu and Lee, 2013[39]). Because of these properties, asbestos is mined and used in manufacturing, especially in the building industry for more than 3000 products, goods, abrasion and heat resistant materials. These properties make asbestos a compressed material in the environment and inseparable. More than 95 % of the world's commercial asbestos is a kind of chrysotile (Kamp and Weitzman, 1999[18]). Mining, milling and processing of asbestos into artificial products make asbestos dust possessing asbestos fibers (Workplace Health and Safety Queensland, 2012[46]). Products of asbestos-cement factory are roofing tiles and asbestos-cement pipes which contain more than 11-13 % chrysotile asbestos (Dopp et al., 2005[8]). Continuous exposure to weather conditions or acid rain, destruct or change the surface of asbestos-cement products resulting in wide spread of asbestos and cement particles in the air and water (Rahman, 1995[34]). The use of asbestos began from the 1950s in Iran; it is widely used in friction materials. Nearly 55,000 tons of chrysotile asbestos entered to Iran in 2007 (Kakooei et al., 2009[16]). Use of asbestos in industrial practices has not declined in recent years, furthermore manufacturing of asbestos products has not ceased yet. Exposure to asbestos is the cause of inflammatory and dangerous lung diseases including asbestosis, lung cancer, mesothelioma and pleural diseases such as pleural plaque. These diseases appear after a period of latency for almost 30 years (Park et al., 2008[30]; Loomis et al., 2010[22]; Seo et al., 2012[40]). In 2005, Iranian Annual of National Cancer Registration Report (IANCRR, 2005[13]) has reported 55 cases of malignant mesothelioma related to asbestos. Generally, there are sufficient evidences of the carcinogenicity of asbestos and also, asbestos has been classified as group 1 carcinogens by International Agency for Research on Cancer (IARC, 1987[14]). Molecular mechanisms involved in the carcinogenic effects of asbestos are still not fully understood. But three main theories have been proposed regarding the pathogenesis of asbestos, which are briefly explained here. 1: The oxidative stress theory remarks that the phagocytic cells due to their inability to fiber digestion generate large amounts of free radicals. In addition, epidemiological studies have identified iron-containing asbestos fibers having more carcinogenic strength. Highly reactive oxygen species (ROS) such as hydroxyl radicals are catalyzed by iron on the surface of the fiber through Fenton-type reactions and play its part in carcinogenicity of asbestos fibers. 2: Chromosome tangling theory argues that asbestos fibers reacted with chromosomes (directly or through the division spindle) and finally cause chromosomal abnormalities. 3: Theory of adsorption of carcinogenic molecules expresses that asbestos fibers in vivo concentrate proteins or chemicals including the components of cigarette smoke (Nagai et al., 2011[28]). Mediated effects of different types of asbestos fibers depend on the fiber size, chemical composition, morphology and surface load. Diseases such as respiratory disorders, asthma and chronic bronchitis have been reported so far in asbestos cement factory workers (Lim et al., 2002[20]). Balachandar et al. (2004[4]) have reported chromosomal abnormalities in factory workers of asbestos-cement. Rahman et al. (1996[36]) have shown chromosomal aberrations, sister chromatid exchanges in peripheral lymphocytes of asbestos-cement factory workers. Another study published by Dušinská et al. (2004[9]) demonstrates DNA and chromosomal damage in the asbestos-cement factory workers. In fact, a raise in free radical production or insufficient antioxidant protection leads to oxidative stress that has an important role in the development of occupational diseases (Pournourmohammadi et al., 2008[33]). Therefore, it is worthy examining if asbestos-cement plant workers are in the risk of toxicity by measuring oxidative stress biomarkers.

## Materials and Methods

Thiobarbituric acid (TBA) (Sigma-Aldrich, Chemie Gmbh, Munich, Germany), trichloroacetic acid (TCA), n-butanol, 2,4,6-tripyridyl striazine (TPTZ), HNO_3_, H_2_SO_4_, HCL, NaCl, PdCl_2_, DTNB (2-nitrobenzoic acid), EDTA, CH_3_OH, Na(SO_4_), sodium hypochlorite, acetic acid, sodium acetate, ethanol from Merck (Tehran, Iran), and human specific ELISA kits for 8-OH-dG from Cayman Chemical Co. (Michigan, USA) were used in this research.

### Study participants

This study is a cross-sectional study involving 50 workers of asbestos-cement organization, having a minimum of 5 years asbestos exposure and also 50 people as control group have been chosen who matched for their age, sex, education and with no occupational history of asbestos exposure with above group. Information, work experience, socio-economic status (income, education) and lifestyle (smoking, alcohol, medicines, vitamins, antioxidant supplements and diet) taken with the help of a questionnaire and every worker was interviewed by a trained interviewer. All patients were under clinical examination to detect signs or symptoms of chronic diseases such as hypertension, heart problems, cancer, thyroid disorders, asthma, diabetes and anemia. People with chronic diseases, alcohol, antioxidants or other drug therapy or exposure to toxic substances and radiation therapy were excluded. This study was approved in the review board of College of Basic Sciences, Tehran Science and Research Branch, Islamic Azad University, Tehran, Iran with code number of 182445. Workers' blood samples were taken before going to work at 7 to 8 a.m. Blood samples collected in heparinized tubes and quickly centrifuged with 3000 rpm (2500 g) for 5 min, and plasma was separated and frozen for further analysis of oxidative stress markers at -80 °C.

### Assay for oxidative stress markers

#### DNA damage

8-hydroxy-2-deoxyguanosine (8-OH-dG) as an index of DNA damage was measured by ELISA kit. 8-OH-dG was measured by an anti-mouse IgG-coated plate and a tracer, which contains an enzyme, a mixture of 8-OH-dG-enzyme and an antibody against 8-OH-dG, both devoid of 8-OH-dG and DNA-incorporated 8-OH-dG (Shigenaga and Ames, 1991[42]).

#### Total thiol molecule

Thiol groups were measured colorimetrically using DTNB. DTNB produces a yellow complex after reacting with these groups, which has a maximum absorption at a 412 nm (Hu and Dillard, 1994[12]).

#### Lipid peroxidation

Malondialdehyde (MDA) levels, an index of lipid peroxidation were estimated by concentration of thiobarbituric acid reactive substances (TBARS) in serum. Serum was mixed with trichloroacetic acid (TCA) (20 %), and the precipitate was dispersed in H_2_SO_4_ (0.05 M). Thiobarbituric acid (0.2 %) and 0.05 M sulfuric acid was added, and the mixture was heated for 30 min in boiling water bath. After cooling the pipes, 4 ml of n-butyl is added, fully blended, and centrifuged in 3000 g for 10 min. LPO adducts is extracted by n-butanol, and the absorbance is measured at 532 nm (Esterbauer and Cheeseman, 1990[10]).

#### Total antioxidant capacity

The basis of measurement of total antioxidant capacity (TAC) was to analyze the ability of plasma in reducing Fe^+3^‏ to Fe^+2^ in the presence of TPTZ. Fe^+2^ ‏-TPTZ as blue complex is absorbed in 593 nm (Benzie and Strain, 1996[5]).

#### Statistical analysis

Data are expressed as mean ± standard deviation (SD). Statistical analyses of the data were analyzed using analysis of variance (One Way ANOVA) and the means were compared by the Tukey's post hoc test. Statistical comparison of abnormal clinical factors between experimental groups was conducted using Chi-square. Pearson correlation coefficient was used to study the association between variables. The results were measured statistically significant in the P < 0.05.

## Results

**Figure 1:**
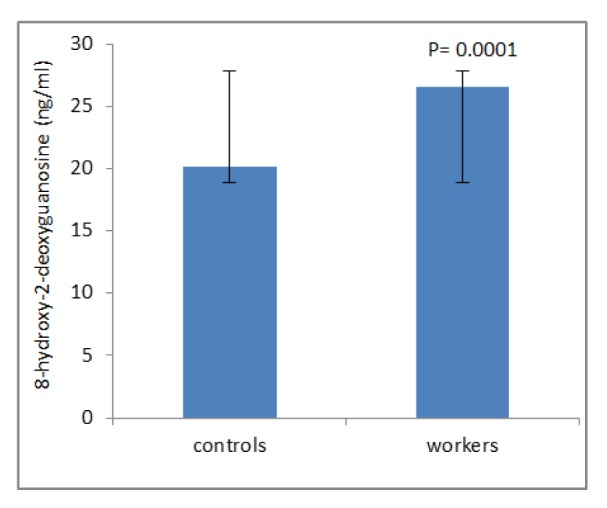
Mean ± SD of 8-hydroxy-2-deoxyguanosine (ng/ml) among workers and controls

**Figure 2:**
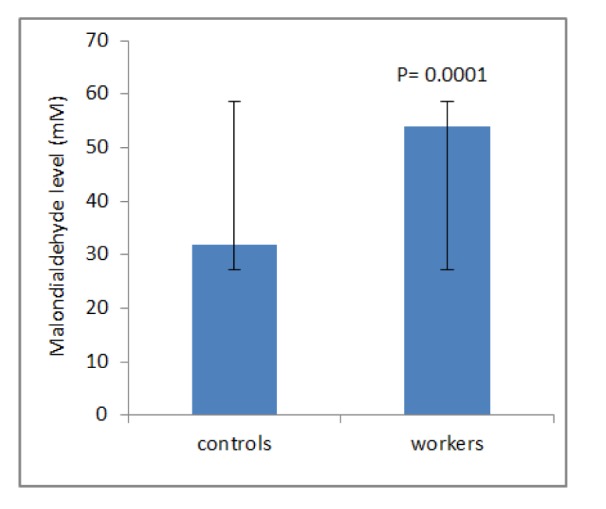
Mean ± SD of malondialdehyde level (mM) among workers and controls

**Figure 3:**
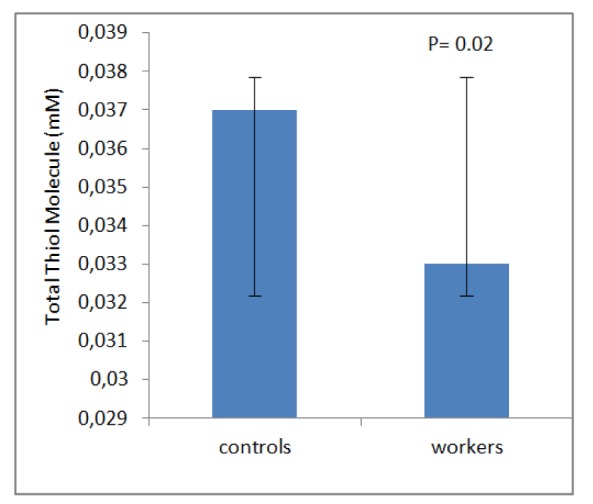
Mean ± SD of Total thiol molecule (mM) among workers and controls group

**Figure 4:**
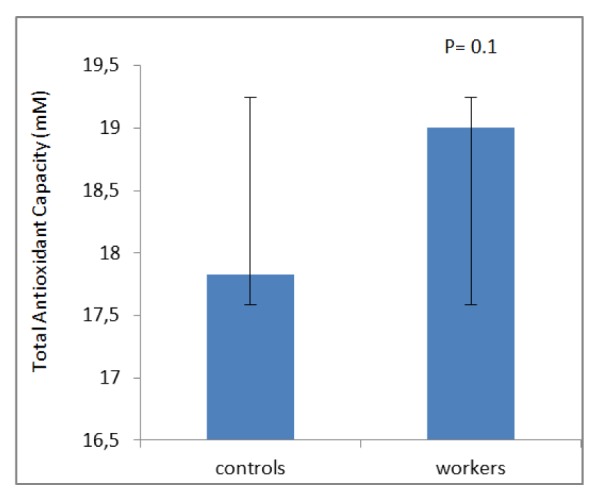
Mean ± SD of Total antioxidant capacity (mM) among workers and controls

Table 1(Tab. 1) shows demographic data of study participants. The mean age of 50 workers that were exposed to asbestos were 36.34 ± 6.82 years, and all of them were male. The control group comprised 50 male workers in the group age of 36.10 ± 7.4. There was no statistically significant difference between the two groups with respect to age, gender and work history. Distribution of smokers and nonsmokers in both groups is depicted in Table 2(Tab. 2). As shown, 28 people of control group were smokers who smoked 11.70 ± 2.75 cigarettes per day. In workers group, 21 of them were smoking 11.40 ± 2.70 cigarettes per day. As can be seen, there was no significant difference (P=0.16) between the number of smokers in both groups. Table 3(Tab. 3) represents the frequency of abnormal clinical findings among the two groups. Compared to the control group, the workers group (irrespective of their smoking habits) had higher prevalence of regular cough, phlegm, wheezing and breathlessness. Additionally, Table 3(Tab. 3) delineates the frequency of abnormal clinical findings among smokers and nonsmokers in the worker and control groups. As depicted, cough and phlegm was seen in 12 and 11 people (57.14 %, 52.36 %, respectively) of the smokers and in 7 (24.13 %) of non-smokers in workers group. Moreover, there was a considerable difference between smokers and non-smokers among workers. Cough and phlegm of the smokers in the asbestos exposed workers was significantly higher than the corresponding subgroup of the control group (smoker in the control group). Wheezing and breathlessness was seen in 6 (28.57 %) of smokers and 4 (13.79 %), 3 (10.34 %), respectively of nonsmokers in worker group. Furthermore, no significant difference in these factors can be seen between smokers and non-smoking workers, while there was a significant difference between smokers in the worker group and smokers of the control group. Table 4(Tab. 4) shows that 8-hydroxy-2-deoxyguanosine and malondialdehyde levels of smokers or non-smokers in the exposed group were significantly higher (p=0.0001) than the corresponding subgroups of the control group. The 8-hydroxy-2-deoxyguanosine and malondialdehyde of the smokers and non-smokers among the asbestos exposed worker group differed considerably (p=0.0001 and p=0.04, respectively). Compared to the smokers in the control group, total thiol molecule of the smokers in the exposed asbestos worker group was significantly lower (p=0.04). There was no major difference between total thiol molecule of the smokers and non-smokers in the exposed asbestos worker group and control group. Overall, no significant difference was seen in total antioxidant capacity between the groups. Correlation analysis demonstrates a significant positive correlation between 8-hydroxy-2-deoxyguanosine and smoking index (N/day) in asbestos exposed workers (r= 0.596, p= 0.0001). Additionally, there is a noticeable positive correlation between malondialdehyde and smoking index (N/day) in asbestos exposed workers (r = 0.403, p= 0.005). It is worthy of notice that no correlation can be seen between 8-hydroxy-2-deoxyguanosine or malondialdehyde and smoking index (N/day) in the control group (r=0.252, p=0.07 and r=0.006, p=0.96, respectively). In addition, correlation coefficient between total thiol molecule or total antioxidant capacity and smoking index (N/day) was statistically insignificant p > 0.05 (r values for the exposed = -0.198 and 0.087, respectively; 0.089 and 0.144, respectively, for the controls). As demonstrated in Figure 1(Fig. 1), the means of plasma 8-hydroxy-2-deoxyguanosine of asbestos exposed workers were significantly higher (p=0.0001) than that of the control group. The means and standard deviation of measurements of 8-hydroxy-2-deoxyguanosine among the exposed and the controls were 26.54 ± 4.99 and 20.17 ± 2.06 ng/ml, respectively. Figure 2(Fig. 2) shows means and standard deviation of malondialdehyde in asbestos exposed workers and the controls. As can be seen the means and standard deviation of malondialdehyde in asbestos exposed workers and the controls were 54 ± 8.31 and 31.83 ± 7.39 mM, respectively, that there was significant difference (p=0.0001) between the two groups. Figure 3(Fig. 3) delineates that the mean of total thiol molecule of asbestos exposed workers was significantly lower (p=0.02) than that of the controls. The means and standard deviation of measurements of total thiol molecule among the exposed and the controls were 0.033 ± 0.009 and 0.037 ± 0.007 mM, respectively. No major difference can be seen (p=0.1) in total antioxidant capacity between asbestos exposed workers and controls as depicted in Figure 4(Fig. 4). The means and standard deviation of measurements of total antioxidant capacity among the exposed and the controls were 19 ± 3.32 and 17.83 ± 3.12 mM, respectively.

## Discussion

The results of the current study indicated that MDA and 8-OH-dG, were significantly higher in workers than the control group. TTM was also significantly lower in workers than the control group and no significant difference observed between the two groups in the TAC. Acute exposure to asbestos is followed by symptoms such as anemia, shortness of breath, cough, phlegm, wheezing, chest pain, weight loss and loss of appetite (ATSDR, 2001[3]). We found that most of the workers who were in contact with asbestos show symptoms of asbestos poisoning. There are some evidences about the role of asbestos-induced oxidative stress and symptoms in individuals who chronically are exposed to asbestos. For example, high levels of 8-oxo-7,8-dihydro-2'-deoxyguanosine (8-oxodG) in workers' blood samples is one of the markers of DNA damage in workers who were exposed to asbestose. In humans, studies have shown high levels of damage of DNA (8-hydroxyguanine adducts and strand fragmentation) and a high frequency of SCE (sister chromatid exchange) in blood cells of workers with occupational exposure to asbestos (particularly chrysotile, and other forms of asbestos like crocidolite) (Marczynski et al., 2000[23][25], 2001[24]). A series of other reports have shown that the rate of (8-oxodG) in urine or blood samples of workers exposed to asbestos in asbestos factory have been high (Tagesson et al., 1993[43]; Takahashi et al., 1994[44]). Abidi et al. (1999[1]) and Afaq et al. (2000[2]) reported production of large amounts of thiobarbituric acid reactive substances (TBARS) and changing in GSH redox system by chrysotile fibers. Another report has shown an increase in TBARS levels in the V79-cells (Chinese hamster lung cells) after 24-48 h of exposure to asbestos-cement (Dopp et al., 2005[8]). Decrease in intracellular GSH levels which observed in incubated cells with chrysotile, could participate in the development of lung disease (Afaq et al., 2000[2]). Mechanisms involved in induced toxicity by asbestos and its’ carcinogenicity are production of reactive oxygen species derived from Fe (ROS) and reactive nitrogen species (RNS) (Rahman et al., 1999[35]). Asbestos has been shown to induce the expression and activity of inducible nitric oxide synthase (iNOS) in alveolar macrophages and mesothelial cells (Kamp and Weitzman, 1999[18]). All types of asbestos contain cation of iron in the part of their crystalination network (amosite, crocidolite, which 27 % of crocidolite is composed of iron) or iron has been considered as a surface contaminant and substitutes Mg^2+^ (chrysotile fiber). Asbestos fibers can generate ROS via Fenton-type reactions catalyzed by existing iron on the surface of the fiber. ROS may be produced during phagocytosis of asbestos fibers by alveolar macrophages or neutrophils (Yoshida et al., 2001[47]). Recently, researchers have found that chrysotile, possibly through hemolysis, can condense iron in surrounding tissues (Maxim and McConnell, 2001[26]). Chrysotile surface contains positive load, while amosite, crocidolite have negative load. Induced hemolysis is a result of the reaction of positive load surface in asbestos with negative sialic acid on cell membranes of RBC. Hemolysis of red blood cells causes the release of hemoglobin and hemoglobin adsorption on the surface of asbestos fibers can participate in production of free radicals induced by asbestos (Nagai et al., 2011[28]). Different capabilities of any type of asbestos regarding the damages on DNA can also partly be explained by their surface load. As mentioned before, chrysotile has a positive surface load. Therefore, chrysotile in surface area provides very suitable surface for biomolecules with negative load like DNA (Pociask et al., 2004[32]). Nagai and coworkers (2011[28]) have reported that asbestos not only adsorb proteins, but also enhance oxidative responses. This claim is also valid with regard to DNA. Asbestos adsorbs DNA and then 8-OH-dG is produced. Combination of 8-OH-dG is a major production of oxidative DNA damage that caused by G → T and A → C substitution. This displacement (substitution) reported as unconscious oncogene places and has been mainly responsible for the initiation of cell proliferation and carcinogenesis (Pociask et al., 2004[32]). Asbestos fibers cause DNA damage in the form of single or double strand breaks (SSBs-DSBs), intra-interstrand cross-linking, and base damage (Roggli et al., 2004[37]). Vital macromolecules such as membrane lipids, DNA and proteins of signal transduction are targets of asbestos and its secondary senders (ROS and RNS) (Rahman et al., 1999[35]). Lipid peroxidation is mediated by asbestos by altering the structure and function of cell membrane (Turci et al., 2012[45]). High level of lipid peroxidation products were found in the plasma of workers exposed to asbestos and have been measured by the TBARS (Kamal et al., 1989[17]). The role of catalyzed ROS by iron, contained in asbestos fiber, in producing lipid peroxidation is suggested by the fact that iron composition alone or as a part of asbestos catalyzes the production of these products and also by the observation that antioxidant such as vitamin E and iron chelators reduces lipid peroxidation (Goodglick et al., 1989[11]). Another finding of the present study is that the TTM has decreased in workers compared to the control group. This has been specifically emphasized by Bhattacharya et al. (2004[6]) who observed that garlic extract, which contains numerous sulfur compounds and glutathione precursors, reduced asbestos-mediated toxicity. In the asbestos exposed group, elevated oxidative markers were observed which were impartially neutralized by TAC, leading to the development of a marked oxidative stress. There was a positive correlation between levels of 8-OHdG and MDA of asbestos workers and the smoking status which refers to the negative role of smoking. There was a positive correlation between the levels of 8-OH-dG or MDA and the smoking index (cigarettes per day) in the asbestos exposed workers group, suggest that DNA damage and MDA may be associated with the number of cigarettes smoked lifetime. Smoking has been most consistently identified as a confounder for 8-OHdG and there was substantial evidence that smokers have higher levels of 8-OHdG and MDA than non-smokers (Pilger and Rüdiger, 2006[31]). Kiyosawa et al. (1990[19]) indicated that cigarette smoking causes oxidative DNA damage in peripheral blood cells. Loft et al. (1992[21]) identify smoking as the most determining factor of the urinary excretion of 8-OHdG, proposing that smoking is associated with a 50 % increase in oxidative DNA damage. It has been reported that smokers have higher plasma MDA concentrations comparing with non-smokers (Shah et al., 2015[41]). Epidemiologic studies have proved tobacco smoke and asbestos exposures synergistically interact to increase lung cancer risk (Nelson and Kelsey, 2002[29]). Cigarette smoking by increasing maintenance of short fibers prevents asbestos clearance. Increased lung fiber burden plays a main role in accelerating diseases rate seen in asbestos workers who smoke (McFadden et al., 1986[27]). Churg et al. (1989[7]) indicated that active oxygen species, perhaps derived from the cigarette smoke, play a role in smoke-mediated fiber transport into tracheobronchial epithelia. This study has identified that chronic occupational exposure to asbestos can lead to increased levels of DNA damage and MDA and reduced TTM and there was a synergistic interaction between smoking and asbestos.

## Conclusion

This study found higher 8-OH-dG and MDA, lower TTM, and similar TAC levels in the experimental groups (i.e. those contacted to asbestos as compared to the controls group). Alteration in oxidative stress markers in the workers can be used as predictive markers for the development of asbestos-related disease such asbestosis, malignant lung cancer, mesothelioma and pleural plaques.

## Acknowledgement

The researchers would like to acknowledge the assistances of all the participants and authorities of the factory that samples were collected. Authors wish to thank Dr Haji Bahadar for his assistance in English language edit of the article.

## Conflict of interest

The author(s) declare no conflicts of interest with respect to the research, authorship, and/or publication of this article.

## Figures and Tables

**Table 1:**
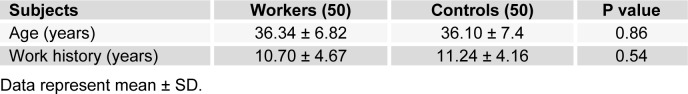
Summary of demographic data in asbestos-cement plant workers and control subjects

**Table 2:**
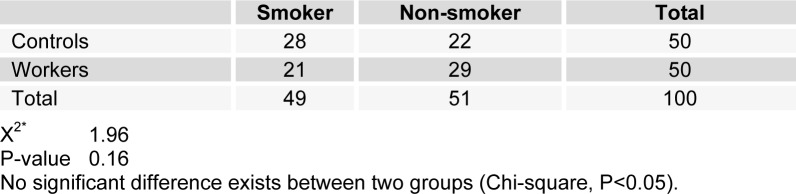
Distribution of study subjects by smoking habits

**Table 3:**
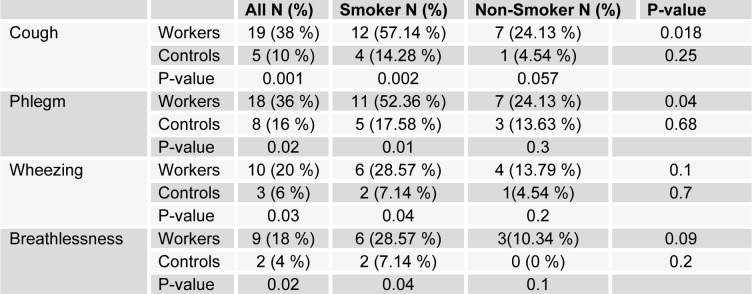
Frequency (%) of abnormal clinical findings in asbestos exposed workers (50) and controls (50), also in the smoker and non-smoker subgroups among asbestos exposed workers and controls

**Table 4:**
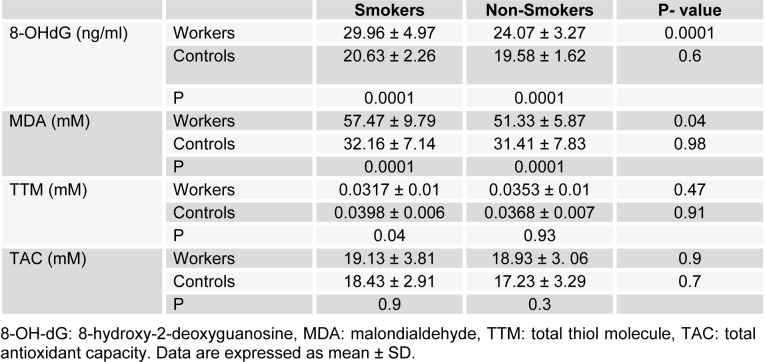
Mean ± SD of Plasma 8-OHdG, MDA, TTM and TAC of smoker and non-smoker subgroups among asbestos exposed workers and controls
